# Unpacking early infant male circumcision decision-making using qualitative findings from Zimbabwe

**DOI:** 10.1186/s12914-016-0111-1

**Published:** 2017-01-09

**Authors:** Webster Mavhu, Karin Hatzold, Getrude Ncube, Shamiso Fernando, Collin Mangenah, Kumbirai Chatora, Roy Dhlamini, Owen Mugurungi, Ismail Ticklay, Frances M. Cowan

**Affiliations:** 1 Centre for Sexual Health & HIV/AIDS Research (CeSHHAR), 9 Monmouth Road Avondale West, Harare, Zimbabwe; 2 University College London, London, UK; 3 Population Services International, Harare, Zimbabwe; 4 Ministry of Health & Child Care, Harare, Zimbabwe; 5 University of Zimbabwe College of Health Sciences, Harare, Zimbabwe

**Keywords:** Decision-making, Early infant male circumcision, Interventions, Qualitative, Zimbabwe

## Abstract

**Background:**

Early infant male circumcision (EIMC) has been identified as a key HIV prevention intervention. Exploring the decision-making process for adoption of EIMC for HIV prevention among parents and other key stakeholders is critical for designing effective demand creation interventions to maximize uptake, roll out and impact in preventing HIV. This paper describes key players, decisions and actions involved in the EIMC decision-making process.

**Methods:**

Two complementary qualitative studies explored *hypothetical* and *actual* acceptability of EIMC in Zimbabwe. The first study (conducted 2010) explored *hypothetical* acceptability of EIMC among parents and wider family through focus group discussions (FGDs, *n =* 24). The follow-up study (conducted 2013) explored *actual* acceptability of EIMC among parents through twelve in-depth interviews (IDIs), four FGDs and short telephone interviews with additional parents (*n =* 95). Short statements from the telephone interviews were handwritten. FGDs and IDIs were audio-recorded, transcribed and translated into English. All data were thematically coded.

**Results:**

Study findings suggested that EIMC decision-making involved a discussion between the infant’s parents. Male and female participants of all age groups acknowledged that the father had the final say. However, discussions around EIMC uptake suggested that the infant’s mother could sometimes covertly influence the father's decision in the direction she favoured. Discussions also suggested that fathers who had undergone voluntary medical male circumcision were more likely to adopt EIMC for their sons, compared to their uncircumcised counterparts. Mothers-in-law/grandparents were reported to have considerable influence. Based on study findings, we describe key EIMC decision makers and attempt to illustrate alternative outcomes of their key actions and decisions around EIMC within the Zimbabwean context.

**Conclusions:**

These complementary studies identified critical players, decisions and actions involved in the EIMC decision-making process. Findings on who influences decisions regarding EIMC in the Zimbabwean context highlighted the need for EIMC demand generation interventions to target fathers, mothers, grandmothers, other family members and the wider community.

## Background

Early infant male circumcision (EIMC, performed within the first 60 days of life) has been identified as a key HIV prevention intervention for sustaining the prevention gains anticipated through adult voluntary medical male circumcision (VMMC) [1–3]. Although its effects on HIV will take longer to realise, EIMC is likely to ultimately be more effective at preventing HIV acquisition than VMMC as the procedure is carried out long before the individual becomes sexually active, negating the risk associated with sex during the healing period [4]. Like VMMC, EIMC will therefore protect against other sexually transmitted infections and genital cancers in addition to HIV [5, 6].

In addition, EIMC is cheaper than VMMC, with studies estimating that it is likely to be a cost-saving HIV prevention intervention in the longer term [7–10]. Projections suggest that providing universal access to male circumcision, including EIMC, in conjunction with other effective HIV prevention interventions, will reduce the overall cost of HIV epidemics driven by heterosexual transmission [7]. Pilot implementation of EIMC is already underway in most of the 14 VMMC priority countries including Botswana, Kenya, Lesotho, Rwanda, South Africa, Swaziland, Tanzania, Uganda, Zambia, and Zimbabwe [11–21].

Zimbabwe has provided VMMC to around 750,000 adult and adolescent men. The program aims to reach 1.3 million 15-29 year-olds by 2017/18. Zimbabwe also intends to offer EIMC alongside VMMC [15, 16, 22]. Since large-scale EIMC for HIV prevention, or indeed for other reasons, has never been practiced in Zimbabwe or more widely in Southern Africa, there is a dearth of literature on EIMC decision-making within this context. However, understanding the EIMC decision-making pathway is crucial for at least two reasons. Firstly, this enables the identification of key players in the decision-making process, who can then be specifically targeted by interventions. Secondly, this allows for the identification of facilitators and barriers associated with each stage of the decision-making process which can then be systematically addressed. Knowing who to target with what information is clearly going to be critical to increasing demand for EIMC. We present results on decision-making around EIMC from two complementary qualitative studies we conducted in Zimbabwe. Findings will inform demand creation during roll out.

## Methods

### Design of studies

As stated earlier, findings reported here are from two complementary qualitative studies. The first study (conducted in 2010) explored *hypothetical* acceptability of EIMC among parents and the wider family through focus group discussions (FGDs) [23]. The follow-up study (conducted in 2013) explored *actual* acceptability of EIMC among parents who had either opted or declined to have their newborn son circumcised. The second study was nested within a trial that assessed the feasibility, safety, acceptability and cost of rolling out EIMC using devices in Zimbabwe, described in detail elsewhere [9, 16, 22]. In both studies, researchers explored among other issues, decision-making around EIMC.

### Sampling and data collection

In the first study, four teams of Zimbabwean researchers (2 male; 2 female) were trained around qualitative data collection and analysis. Between June and October 2010, the four teams conducted a qualitative study with rural and urban participants in five of Zimbabwe’s 10 provinces: Bulawayo, Harare, Mashonaland West, Masvingo and Matebeleland North. Twenty-four gender-specific FGDs were held with expectant mothers (*n =* 6 groups), expectant fathers (*n =* 5 groups), grandfathers/fathers-in-law (*n =* 7 groups), grandmothers/mothers-in-law (*n =* 6 groups).

In the second study, two teams of trained and experienced Zimbabwean researchers (1 male; 1 female) held a predetermined number of gender-specific in-depth interviews (IDIs) and FGDs in Harare with parents. The numbers included were in part limited by budgetary and time constraints. Between January and May 2013, six in-depth interviews and two FGDs were held with parents who had adopted EIMC for HIV prevention (*n =* 3 IDIs and 1 FGD with 10 mothers; *n =* 3 IDIs and 1 FGD with 9 fathers). Participants for these IDIs and FGDs were randomly selected from a list of parents that had adopted EIMC. A further six in-depth interviews and two FGDs were held with parents who had declined to circumcise their newborn sons (*n =* 3 IDIs and 1 FGDs with 10 mothers; *n =* 3 IDIs and 1 FGD with 9 fathers). Participants for these IDIs and FGDs were randomly selected from a list of couples that had not adopted EIMC, and had not been shortlisted for short phone interviews.

Furthermore, short phone interviews (*n =* 95) were conducted with parents who had arranged to bring their sons for EIMC but then defaulted. The phone interviews only sought to ascertain their reasons for not bringing the infant for the procedure. In these phone interviews, we included 15 out of the 17 parents (88%) who had gone through all study screening procedures (including providing locator information, comprehension of screening eligibility criteria and responding to a one-time questionnaire) but did not eventually enrol. Of these 15, ten were mothers; five were fathers. The other 80 phone interview participants were selected as follows: 65 females (10%) were randomly selected from a list of 650 mothers who had arranged to bring their sons for EIMC but then defaulted. An additional 15 males (10%) were randomly selected from a list of 150 fathers who had participated in weekend group meetings and promised to bring their sons for EIMC but then defaulted. Phone interview participants were contacted when their sons were no longer eligible for EIMC. Short statements were handwritten at the time of the call and later coded.

All discussions for both studies were conducted in either Shona or Ndebele, Zimbabwe’s dominant indigenous languages, also spoken and understood by smaller ethnic groups. FGDs lasted 2–2.5 h whilst in-depth interviews lasted 45 min to one hour. All FGDs and in-depth interviews were audio-recorded.

### Data analysis

Audio-recorded qualitative data were transcribed and translated verbatim into English. Initial themes were identified during the data collection process; these were used to develop an initial coding framework. Additionally, for both studies, five in-depth interviews and four FGDs were then coded line by line on paper by two researchers using the coding framework. Additional codes were added to the coding framework. Transcripts were then entered into NVivo (QSR International, Melbourne, Australia), a qualitative data storage and retrieval program. For both studies, two trained and experienced researchers coded each transcription separately using the modified coding framework, taking note of any emerging new codes. If there were disagreements over the interpretation of some codes, the senior social scientist (WM) met with the two researchers. The three would examine the codes and collectively agree on the standard forms to use for coding; the coding framework was subsequently revised in line with any agreed changes. WM checked concordance of the two researchers’ coding in addition to independently coding all transcripts.

Handwritten statements obtained from the phone interviews were typed and entered into an excel document. Researchers read the statements and assigned each statement a code based on its key words. Codes were grouped into categories and emerging themes were then identified following the principles of thematic analysis [24, 25]. During write-up, themes and sub-themes were illustrated with verbatim quotes.

## Results

A total of 240 participants aged 18–80 years took part in the 24 FGDs of the first study which explored *hypothetical* acceptability of EIMC. Of the 240 participants 130 (54%) were female. Fourteen FGDs were held in urban areas and 10 in rural communities. Thirty-seven parents of newborn male infants (*n =* 20 mothers; *n =* 17 fathers) took part in the four FGDs of the second study. Also, 12 in-depth interviews were held with parents of newborn male infants (*n =* 6 mothers; *n =* 6 fathers). An additional 95 parents took part in short telephone interviews (*n =* 75 mothers; *n =* 20 fathers). Based on study findings, we describe key EIMC decision makers and attempt to illustrate alternative outcomes of their key actions and decisions around EIMC.

### Decision-making: infant’s parents

In the first study, which explored *hypothetical* acceptability of EIMC, when parents were asked to envisage who would be involved in the process of deciding about EIMC, younger participants (expectant parents) felt that this would involve a discussion between the infant’s parents. *‘The decision that matters is that of the father and the mother; they sit down and talk about it’* (expectant mother, 1^st^ study fgd16). An expectant father concurred, ‘*You discuss it together with the mother [infant*’*s*] *and you reach a consensus*’ (expectant father, 1^st^ study fgd10). In the second study, which explored *actual* acceptability of EIMC, accounts surrounding EIMC uptake also suggested that the decision-making process had involved a discussion between the infant’s parents. A mother described events leading to their son’s circumcision.

…*I listened to a lady who talked about EIMC when I was still pregnant. I took a pamphlet she had given me home*, *gave it to my husband and we discussed the issue. My husband said that it was a good thing. He said if I gave birth to a baby boy*, *we were supposed to take him for circumcision immediately after birth. When I gave birth to a baby boy*, *I simply went ahead* … (mother, 2^nd^ study IDI5).

Some male participants also stated that the decision to circumcise their son had involved some discussion. ‘*In my case we discussed the issue since we had never heard of it* [*EIMC*]. *We agreed that our son might benefit*… *it was an* “*experiment*”…’ (father, 2^nd^ study fgd1). Another male participant described the danger associated with making a unilateral decision in respect of newly-introduced initiatives. ‘…*Especially for something that is new*, *if you want to exercise your powers on something you don*’*t know*, *you will end up shouldering the blame*’ (father, 2^nd^ study fgd1). It appears that as EIMC was a new initiative, men did not feel confident about overriding their wife’s decision in the way that they might for other decisions.

Nonetheless, in both studies, male and female participants of all age groups acknowledged that the father has the final say. ‘*The man must make that decision because he is the one who knows whether or not that is practiced in his clan*; *a woman cannot know anything about a clan to which she doesn*’*t belong*’ (father-in-law, 1^st^ study fgd18). The implication here is that if male circumcision is not practiced in the father’s clan it would not be advisable to circumcise the male infant. A female participant concurred, ‘*As the mother*, *I cannot decide whether or not the child should be circumcised. I will need to* “*sit down*” [*discuss*] *with the father and we will have to go by his decision*’ (expectant mother, 1^st^ study fgd3). This sentiment was echoed by one father, ‘*The decision to circumcise my son rests with me. I*’*m the one who tells the wife what to do*’ (father, 2^nd^ study IDI4).

Even though the father was deemed to have the final say, discussions around EIMC uptake suggested that mothers of newborn babies took advantage of men’s anxiety about decision-making to influence the outcome. As one mother described,

*If you were not interested* [*in EIMC*] *you could lie to him [baby’s father] that a neighbour had her son circumcised and something undesirable happened. If you were interested, you could also lie to him that several neighbours had their sons circumcised and everything went on well* (mother, 2^nd^ study fgd3).

Another mother described how she had successfully threatened her husband. *‘I told him that I would go to the rural areas and leave him with the child if he went ahead with the circumcision and he backtracked’* [laughter] (mother, 2^nd^ study fgd4).

### Decision-making: wider family

Although both studies suggested that the infant’s parents are the key decision makers, subsequent probing suggested that mothers-in-law/grandparents are also likely to have considerable influence. In the first study, when elderly men were asked what they would do if a daughter-in-law turned down a suggestion to circumcise her son, one of them questioned, ‘*Where does the [infant’s] mother fit in?’* (grandfather, 1^st^ study fgd19). He went on to proclaim, ‘*The mother of the child is also my child’* (grandfather, 1^st^ study fgd19). This sentiment was echoed by an older woman. *‘The daughter-in-law will not refuse; I have powers over the grandchild…I will take him for circumcision myself’* (mother-in-law, 1^st^ study fgd21). Discussions also suggested that the wider family’s influence is sometimes covert. An expectant mother described steps she would take if her husband refused to have their son circumcised if that was what she wanted. *‘If he [father] refuses, I will talk to his mother and she will then ask his uncles to talk to him’* (expectant mother, 1^st^ study fgd14).

Findings from the subsequent study corroborated those from the earlier one with regards to the role of the wider family in the EIMC decision-making process. Parents stated that they had consulted other family members prior to adopting EIMC. Barring the decision by one or both parents, the mother-in-law’s perspective was instrumental in adoption or non-adoption of EIMC. A mother who did not adopt early infant male circumcision described how her husband’s mother had shot down her proposal to adopt EIMC. *‘She said, “I have never heard of it. All of my sons are uncircumcised and even my husband wasn’t circumcised”’* (mother, 2^nd^ study IDI8). Responses to short telephone interviews confirmed FGD and in-depth interview findings with regards to the mother-in-law’s centrality in the EIMC decision-making process. Of the 18 instances where telephone interview respondents mentioned the actual person who had said the male infant should not be circumcised, in 50% of the cases it was the infant’s father, followed by the infant’s mother (28%) and the mother-in-law (22%).

Mothers-in-law did not always block EIMC; they were sometimes quite supportive. A male participant described how his mother had supported EIMC.

*…She really welcomed the idea because she has got nursing friends so she knows that HIV is resulting in this and that. So she was quite supportive; she even asked what I was thinking about our first child. He is three years old now and she said, ‘What are you going to do with this one?’ So she had no problems with us circumcising the newborn* (father, fgd1).

It appears that if the mother-in-law had some MC and HIV knowledge, she was likely to encourage her son and daughter-in-law to adopt EIMC.

### Decision-making: process

**Fig. 1:**
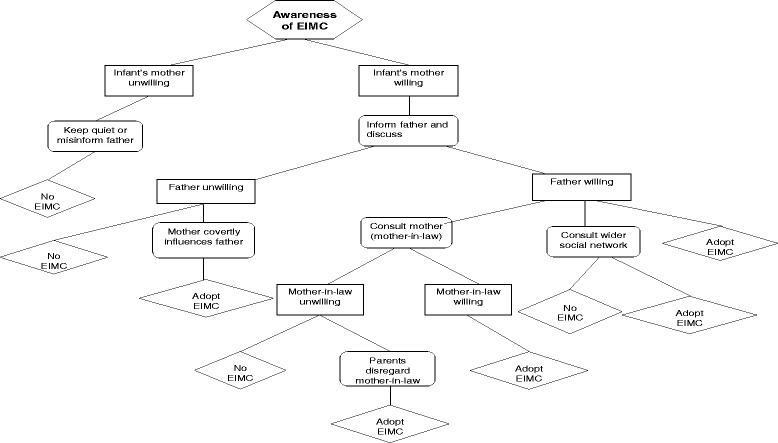
Zimbabwe EIMC decision tree

Triangulating findings from the two studies described here, we have come up with an EIMC decision tree in an attempt to illustrate the most commonly described decision-making process uncovered during discussions. We present key decision makers as well as alternative outcomes of their key actions and decisions around EIMC within the Zimbabwean context. In summary, based on study findings, we have illustrated that when the mother becomes aware of the possibility of EIMC for her newborn son, she either keeps quiet or misinforms the infant’s father about the risks of EIMC if she does not want to have her baby circumcised. On the other hand, if she wants to have her son circumcised, she informs the father about the possibility of EIMC and a discussion of the pros and cons ensues. If the father objects to EIMC, the baby will not be circumcised (unless the infant's mother covertly influences EIMC adoption). However if the father is interested, he will consult his own mother (wife’s mother-in-law) and/or wider social support network whose views may or may not influence the ultimate outcome (Fig. 1). The main parental motivator for adopting EIMC was the desire to protect son from future sexually acquired HIV infection. Of note, most of the men who adopted EIMC reported having undergone voluntary medical male circumcision themselves. Parental reasons for non-adoption of EIMC included fear of harm and socio-cultural considerations [22, 26].

## Discussion

We utilized data from two complementary qualitative studies to unpack EIMC decision-making within the Zimbabwean context. This enabled us to identify key decision makers as well as alternative outcomes of their key actions and decisions around EIMC.

Data highlight the crucial role fathers play in EIMC decision-making. These findings are consistent with those from other settings [27–30]. A study conducted in Mysore, India also found that the father has the final say in whether the infant is circumcised or not [27]. Within sub-Saharan Africa, the findings are consistent with those from Western Kenya and Zambia [28, 30]. In the Kenyan study, fathers of babies who had undergone EIMC were the primary decision makers in most instances, according to interviews with mothers and fathers [30]. A qualitative study conducted in Zambia also found that the father had the final say with regards to EIMC decision-making [28]. In that study, among women who had accepted EIMC, most said their husbands had ‘authorized’ them to take along their sons for the procedure while the majority of women in the groups that decided against EIMC said it was their husbands who had ultimately refused [28]. An exception is the Botswana case where the majority of women (63%) identified themselves as primary decision makers [4], likely explained by the fact that marriage is much less ubiquitous in Botswana than in Zimbabwe. Within the sub-Saharan African context, unmarried women have more autonomy with regards decision-making than married ones.

On the whole, the findings from the studies presented here, coupled with those from other settings, suggest that since fathers make the ultimate decision as to whether their infant son should be circumcised or not, they need to be provided with information *directly* not just through their wives. Given that men are notoriously hard to reach via health services [31–33], other venues for information sharing need to be considered. Interestingly, most of the men who adopted EIMC reported having undergone VMMC themselves. A study on actual EIMC acceptability conducted in Kenya also found that the circumcision status of the infant’s father was associated with increased likelihood of EIMC adoption [30]. These findings and those from our study suggest that as adult VMMC becomes more prevalent, demand for EIMC is likely to increase. Consequently, men who undergo VMMC need to be sensitized on both the availability and comparative advantages of EIMC.

An attempt to map the EIMC decision-making process — especially within the context of a research initiative — is extremely challenging. Despite adult VMMC scale-up in the 14 priority countries over the last 4–5 years, researchers have not yet managed to adequately dissect the adult VMMC decision-making process. Some research groups have attempted to do this and have identified some of the important factors while acknowledging that their understanding of the process is incomplete [34–36]. They have, however, identified a range of motivators, including those unrelated to risk of HIV including improved hygiene, perceptions of responsible masculine choice, perceptions of sexual partner preferences, and improved health for female sexual partners (e.g. reduced risk of cervical cancer) [34, 36].

By better understanding the motivating factors, messages to influence uptake of adult VMMC have been redesigned. For example, VMMC in Zimbabwe has been repackaged as a lifestyle choice rather than an HIV prevention method so as to increase acceptance of the service by both men and women, in addition to countering perceptions that the procedure only benefits “promiscuous” men [37]. This has coincided with increased uptake of VMMC across the country and anecdotal evidence suggests that this repackaging is making VMMC more acceptable to women.

Despite the difficulty in unpacking the VMMC decision-making process, it is generally accepted that decision-making is characterized by an interplay of various factors at the individual, household and community levels [38]. Additionally, factors that influence or hinder male circumcision uptake in general and EIMC specifically, are to a large extent, context specific [34, 38]. It may therefore be difficult to come up with a description of decision-making that can be applied across the region or contexts. Nonetheless, our findings which identify key decision makers as well as alternative outcomes of their key actions and decisions around EIMC, will likely be useful in informing targeted demand creation initiatives.

Data presented here are from two complementary studies that explored both *hypothetical* and *actual* EIMC decision-making. That there was concordance between data obtained from the two studies points to the likely validity of the findings presented here. Also, the studies triangulated various data collection methods (FGDs, in-depth interviews and short phone interviews). With specific reference to qualitative research, triangulation has been widely adopted as a means of investigating the validity of both the data and the conclusions derived from them [24]. In this case, there was concordance among data obtained through FGDs, in-depth interviews and short phone interviews, highlighting not only the likely validity of the results but also the value of triangulation.

A potential limitation is that for the second study, we conducted a predetermined number of in-depth interviews (*n =* 12), FGDs (*n =* 4) and short telephone interviews (*n =* 95) based on pragmatic considerations. It could be argued that parents with newborn sons who declined to participate in the EIMC trial (*n =* 984) were not adequately represented in this relatively small qualitative piece. Also, it is possible that we did not manage to adequately map the EIMC decision-making process especially since we attempted to do this within the context of a research initiative.

## Conclusion

The complementary studies described here enabled us to explore both hypothetical and actual EIMC decision-making among parents and the wider family. Findings will likely be useful in informing targeted demand creation initiatives to support wider adoption of EIMC. Although issues related to EIMC decision-making are to some degree context specific, some of those identified in these studies may apply in other regional settings intending to roll out EIMC.

## Abbreviations

EIMC: Early infant male circumcision
FGD: Focus group discussion
IDI: In-depth interview
VMMC: Voluntary medical male circumcision
